# Dissociable Functional Brain Networks Associated With Apathy in Subcortical Ischemic Vascular Disease and Alzheimer’s Disease

**DOI:** 10.3389/fnagi.2021.717037

**Published:** 2022-02-03

**Authors:** Sabri Altunkaya, Sheng-Min Huang, Yen-Hsuan Hsu, Jir-Jei Yang, Chien-Yuan Lin, Li-Wei Kuo, Min-Chien Tu

**Affiliations:** ^1^Department of Electrical and Electronics Engineering, Necmettin Erbakan University, Konya, Turkey; ^2^Institute of Biomedical Engineering and Nanomedicine, National Health Research Institutes, Miaoli, Taiwan; ^3^Department of Psychology, National Chung Cheng University, Chiayi, Taiwan; ^4^Center for Innovative Research on Aging Society (CIRAS), National Chung Cheng University, Chaiyi, Taiwan; ^5^Department of Radiology, Taichung Tzu Chi Hospital, Buddhist Tzu Chi Medical Foundation, Taichung, Taiwan; ^6^GE Healthcare, GE Medical Systems Taiwan, Ltd., Taipei, Taiwan; ^7^Institute of Medical Device and Imaging, National Taiwan University College of Medicine, Taipei, Taiwan; ^8^Department of Neurology, Taichung Tzu Chi Hospital, Buddhist Tzu Chi Medical Foundation, Taichung, Taiwan; ^9^Department of Neurology, School of Medicine, Tzu Chi University, Hualien, Taiwan

**Keywords:** functional magnetic resonance imaging, resting-state functional connectivity, disconnection syndrome, apathy, subcortical ischemic vascular disease, Alzheirmer’s disease

## Abstract

Few studies have investigated differences in functional connectivity (FC) between patients with subcortical ischemic vascular disease (SIVD) and Alzheimer’s disease (AD), especially in relation to apathy. Therefore, the aim of this study was to compare apathy-related FC changes among patients with SIVD, AD, and cognitively normal subjects. The SIVD group had the highest level of apathy as measured using the Apathy Evaluation Scale-clinician version (AES). Dementia staging, volume of white matter hyperintensities (WMH), and the Beck Depression Inventory were the most significant clinical predictors for apathy. Group-wise comparisons revealed that the SIVD patients had the worst level of “Initiation” by factor analysis of the AES. FCs from four resting state networks (RSNs) were compared, and the connectograms at the level of intra- and inter-RSNs revealed dissociable FC changes, shared FC in the dorsal attention network, and distinct FC in the salient network across SIVD and AD. Neuronal correlates for “Initiation” deficits that underlie apathy were explored through a regional-specific approach, which showed that the right inferior frontal gyrus, left middle frontal gyrus, and left anterior insula were the critical hubs. These findings broaden the disconnection theory by considering the effect of FC interactions across multiple RSNs on apathy formation.

## Introduction

Apathy is defined as a “lack of motivation that is not attributable to a diminished level of consciousness, cognitive impairment, or emotional stress” (Marin, 1991). Apathetic patients often exhibit diminished self-initiated action to outer stimuli and are unable to initiate purposeful behavior. Apathy is one of the most common behavioral and psychological symptoms of dementia (Kaufer et al., 2000; Lyketsos et al., 2002), and it can be debilitating when it becomes resistant to treatment. Such behavioral and cognitive inertia not only hinders social engagement, but also worsens functional disability which inevitably leads to early institutionalization and increased caregiver stress (Landes et al., 2001). Aside from external factors such as socioeconomic status and environmental factors (Brodaty and Burns, 2012), interdisciplinary research has reported potential associations between apathy and several cognitive processes, including an impaired teleceptive sensory system (Duffy, 2000), executive dysfunction (Duffy, 2000), dysregulated auto-activation (Quaranta et al., 2012), and emotional blunting (Duffy, 2000). Previous studies also supported the multi-dimensional nature of the 18-item Apathy Evaluation Scale (AES), as it can be categorized into subscales or symptom clusters, such as “lack of insight”, “motivation”, “awareness”, and “task completion”, depending on the literatures (Marin et al., 1991; Clarke et al., 2007; Hsieh et al., 2012; Guercio et al., 2015). Moreover, apathy has been conceptualized to include several dissociable dimension or subtypes such as social or emotional apathy in addition to primary motivation deficits (Robert et al., 2009; Ang et al., 2017). As apathy manifestations can vary across dementia subtypes (Tu et al., 2017a), it is important to characterize the features of apathy and examine potential group effects. Therefore, factor analysis by psychological metrics in addition to parceling neuroimaging metrics can provide important clinical information, which could assist in managing apathy with a disease-specific paradigm in addition to a more general concept.

Alzheimer’s disease (AD) is the leading cause of dementia worldwide (Rizzi et al., 2014; Nichols et al., 2019). Vascular cognitive impairment (VCI) is the second most common cause of dementia (Rizzi et al., 2014), and subcortical ischemic vascular disease (SIVD) is the most common cause of VCI (Kalaria, 2016; Dichgans and Leys, 2017). Compared to patients with AD, patients with VCI, and especially those with SIVD (Tu et al., 2017a), are prone to exhibit apathy along with the decline of dementia stages. Due to the pathological hallmarks including confluent white matter hyperintensities (WMH) and/or lacunes, profound apathy in SIVD is regarded to be one of the most detrimental consequences of vascular damage within subcortical structures (Hollocks et al., 2015; Tu et al., 2017a). Neuroimaging techniques such as magnetic resonance imaging (MRI) can provide valuable insights into *in vivo* pathological changes as well as their clinical relevancy among demented patients. Several studies have reported that brain parenchyma changes visualized through various MRI techniques were related to apathy formation among patients with dementia (Lavretsky et al., 2008; Kim et al., 2011; Tunnard et al., 2011; Yi et al., 2012; Tu et al., 2017a). For instance, structural MRI of AD patients has related apathy with volume reduction within the anterior cingulate cortex, lateral orbitofrontal cortex, and superior and ventrolateral frontal regions of the left side (Tunnard et al., 2011). In addition, studies on diffusion tensor imaging (DTI) measurements, which can reflect microstructural changes, have indicated that changes in fractional anisotropy within the left anterior cingulum (Kim et al., 2011) and left superior longitudinal fasciculus (Tu et al., 2017a) were associated with apathy formation in patients with AD. With regards to studies on SIVD, alterations in volumetric quantification including reductions in gray (Yi et al., 2012) and white matter volume (Lavretsky et al., 2008), greater lacune volume, and smaller hippocampal volume have been related to apathy severity (Lavretsky et al., 2008). Furthermore, studies using DTI have revealed that microstructural changes within the right superior longitudinal fasciculus, right inferior longitudinal fasciculus, and bilateral forceps minor could significantly predict apathy severity (Tu et al., 2017a). Given that brain tissue of patients with SIVD has a greater vascular burden primarily located within the subcortical structures compared to patients with AD (Tu et al., 2017a,^2021^), the “disconnection syndrome” has been hypothesized to play an important role in apathy formation among those burdened by cerebral vascular pathology (Tu et al., 2017a; Le Heron et al., 2018; Tay et al., 2019). The main concept of disconnection syndrome elaborates the mechanism that damaged association and/or commissural fibers originally relay critical connections amongst remote regions to create a coordinated and intact cognitive process (Catani and Ffytche, 2005). Defective integrity of these important connections thus triggers the disconnection of both directly and indirectly connected remote cerebral regions. Therefore, characterizing the connectivity among these remote brain regions will provide insight into the formation of apathy.

Resting state functional MRI (rs-fMRI) can be used to evaluate cerebral functional connectivity (FC) by measuring time-varying neuronal activity in spatially remote brain regions (Biswal et al., 1995). Several studies have addressed FC alterations in relation to apathy among AD spectrum disorders (Zhao et al., 2014; Büyükgök et al., 2020; Tumati et al., 2020). A recent rs-fMRI study reported that apathy was associated with a marginal decrease in default mode activity within the anterior cingulate cortex and its associated FC networks (Büyükgök et al., 2020). Reduced nodal density, which is reflected as lower local efficiency and cluster coefficient, has been associated with apathy formation (Tumati et al., 2020). In a task-dependent fMRI experiment, reduced functional activity within the bilateral amygdala to visual signals with negative emotional valence was also recorded among AD patients with apathy compared to those without apathy (Zhao et al., 2014). Although some studies have reported a relationship between FC and apathy in AD patients, this relationship among patients with SIVD remains undetermined. Several rs-fMRI studies have attempted to investigate the association between SIVD pathology and the disconnection process by characterizing local or global FC. Locally, decreased regional homogeneity of rs-fMRI signals within the right middle frontal gyrus and left anterior cingulate gyrus in relation to attention deficits has been identified in patients with SIVD (Tu et al., 2020). Globally, FC alterations within the default-mode network and right fronto-parietal network have been reported among subjects with WMH (Liang et al., 2016), and the FC could partly decrease (Sang et al., 2018) or vary in pattern (Zhang et al., 2013) as cognition declined. Another study also described a mosaic pattern of FC changes in SIVD, in which both decreased thalamic FC and also increased FC in some frontal regions were found (Zhou et al., 2016). However, few studies have investigated the difference in FC between SIVD and AD, and knowledge about the association of FC within multiple resting state networks (RSNs) with apathy is particularly limited. Therefore, the aims of this study were to (i) structuralize apathy formation according to clinical and psychological factors, (ii) identify differences in FC between patients with SIVD and AD by characterizing the RSNs, and (iii) explore the association between FC and apathy formation at intra- and inter-RSN levels.

## Materials and Methods

### Participants and Inclusion/Exclusion Criteria

Twenty-three patients with SIVD, 34 with AD, and 23 participants with normal cognition (NC) were recruited in this study. All patients in the SIVD and AD groups had cognitive complaints and a Mini-Mental State Examination (MMSE) (Shyu and Yip, 2001) score ≤ 26. All participants in the NC group were free from cognitive complaints, and their MMSE (Shyu and Yip, 2001) scores were all > 26. The inclusion and exclusion criteria for SIVD and AD were the same as described in previous studies (Tu et al., 2017b,^2020^; Supplementary Table 1). In addition to research criteria for SIVD (Erkinjuntti et al., 2000) and the National Institute on Aging-Alzheimer’s Association Criteria (McKhann et al., 2011) for AD, the inclusion criteria for the SIVD and AD groups in this study were a Hachinski Ischemic Scale (HIS) score (Hachinski et al., 1975) ≥ 7 and ≤ 4, respectively. It is generally accepted in clinical practice that the possibility of mixed dementia can be lowered by using the HIS (Hachinski et al., 1975). This study was approved by the Institutional Review Board at our hospital (#REC 106-09) and informed consents of all participants were well received.

### Clinical Data Registry and Screening of Neuropsychological Symptoms

The demographics, social background, vascular factors, and global cognition of all participants were recorded. Total HIS and Fazekas Scale scores were used to assess systemic and cerebral vascular factors, respectively. The Fazekas Scale scoring was rated by a neurologist (MCT; 15-year clinical experience) who followed the scoring standard (Supplementary Table 2). Cognitive tests and evaluations of neuropsychological symptoms were all conducted by certified clinical psychologists. To sufficiently document global cognition status, both the MMSE and Cognitive Abilities Screening Instrument were administered, with a higher score indicating better cognition. In addition, the Clinical Dementia Rating (CDR) scale was used to evaluate cognitive performance, with a higher score indicating higher dementia severity. The CDR sum of box (CDR_SOB) score was calculated by summing each CDR domain, and it was used for statistical analysis due to its primarily non-parametric property (Morris, 1993).

The screening tools used to evaluate neuropsychological symptoms included the Neuropsychiatric Inventory (NPI) (Cummings et al., 1994; Kaufer et al., 2000), Beck Anxiety Inventory (BAI) (Steer and Beck, 1997), and Beck Depression Inventory (BDI) (Beck et al., 1996). The NPI is a semi-structured interview focusing on both the severity and frequency of disturbances according to 12 behavioral and psychological symptoms of dementia (Kaufer et al., 2000). The BAI (Steer and Beck, 1997) and BDI (Beck et al., 1996) were primarily used to detect profound anxiety and depression, which can potentially interact with apathy (Marin et al., 1991). Both the patients and their respective informant’s reports were taken into consideration. A higher score on these screening tools indicated a greater degree of corresponding symptoms.

### Apathy Evaluation Scale-Clinician Version

The AES, clinician version, is a questionnaire containing 18 items scored using a 4-point Likert scale (Marin et al., 1991). To characterize apathy, the scores for each item are based on the degree that is characteristic for the corresponding description using both verbal and non-verbal information. Ratings were based on the situation over the past 4 weeks, with a higher score representing greater apathy (Lueken et al., 2007; Hsieh et al., 2012). The total score of the Apathy Evaluation Scale (AES_T) was calculated by summing the scores of all individual items. The reliability of the AES and its translated version have both been validated (Marin et al., 1991; Hsieh et al., 2012).

### MR Experiments

MR experiments were performed on a 3 T MRI scanner (Discovery MR750, GE Medical Systems, Milwaukee, WI) with an 8-channel phased-array head coil. Three kinds of MR protocols were acquired, including 3-dimensional T1-weighted imaging (3D-T1), T2 fluid-attenuated inversion recovery imaging (T2-FLAIR), and rs-fMRI. A spoiled gradient echo with RF spoiling scheme (GE FSPGR) was used for 3D-T1 image acquisition with a repetition time (TR) of 7.904 ms, echo time (TE) of 3.06 ms, inversion time (TI) of 450 ms, flip angle of 12°, field-of-view (FOV) of 240 mm, matrix size (MTX) of 240 × 240 × 160, and isotropic voxel size of 1 mm^3^. For T2-FLAIR, the sequence parameters were a TR of 12,000 ms, TE of 120 ms, TI of 2,200 ms, FOV of 220 mm, MTX of 384 × 224, slice thickness (SL) of 5 mm, and 21 slices. The T2-FLAIR images were used to semi-quantify WMH according to the Fazekas scale (Fazekas et al., 1987). Gradient echo echo-planar-imaging (EPI) was used for rs-fMRI data acquisition with a TR of 2,500 ms, TE of 30 ms, flip angle of 90°, FOV of 200 mm, MTX of 64 × 64, SL of 3 mm, and 47 slices. A total of 154 volumes of EPI images were obtained.

### Pre-processing of rs-fMRI Data

**FIGURE 1:**
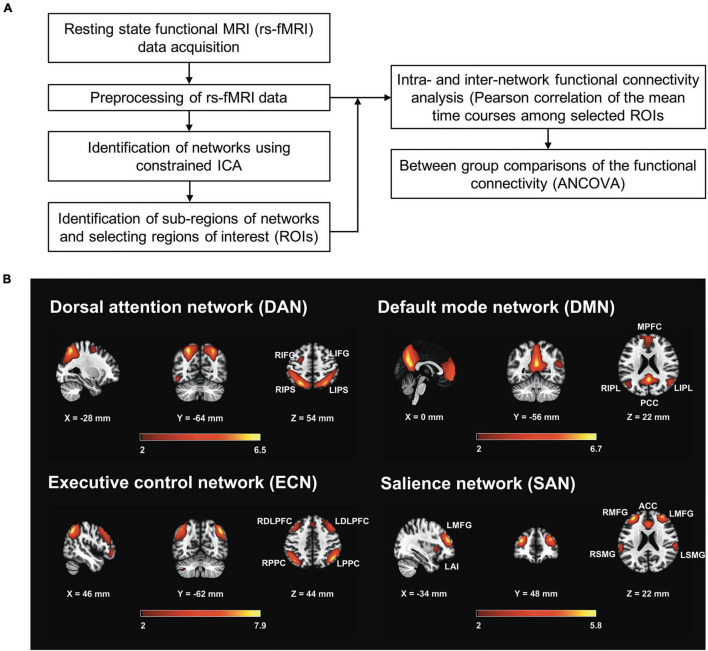
Work flow of imaging processing and related statistics in the current study. **(A)** The clusters of the pre-processed resting-state functional MRI (rs-fMRI) data were extracted by searching local maximum of t-map of mean ICA components. Major clusters were chosen according to resting-state networks (RSNs) defined in the literatures and then used for serving as the region-of-interests (ROIs). Each ROI was defined as a spherical region with the radius of 6 mm centering at the voxel of local maximum. The rs-fMRI time series intra each ROI was averaged and used for analyses of intra- and inter-network functional connectivity. Comparison by group was assessed by analysis of covariance (ANCOVA) with controlling for age, education, and global cognition. **(B)** Four RSNs, including the dorsal attention network (DAN), default mode network (DMN), executive control network (ECN), and salience network (SAN) using the constrained group independent component analysis were identified. Z-score maps (*z* > 1.96) of all four brain networks and their sub-regions are displayed. DAN: L/RIPS, left/right inferior parietal sulcus; L/RIFG, left/right inferior frontal gyrus; DMN: MPFC, medial prefrontal cortex; PCC, posterior cingulate cortex; L/RIPL, left/right Inferior parietal lobule. ECN: L/RPPC, left/right posterior parietal cortex; L/RDLPFC, left/right dorsolateral prefrontal cortex. SAN: ACC, anterior cingulate cortex; L/RMFG, left/right middle frontal gyrus; L/RSMFG, left/right supramarginal gyrus; LAI, left anterior insula (to be the representative for template illustration).

Figure 1 illustrates the workflow of image processing and data analysis conducted in this study. The pre-processing and denoising procedures were carried out using the functional connectivity toolbox CONN version 19.c.^1^ The pre-processing steps included functional image realignment and unwarping, slice-timing correction, outlier identification, direct segmentation, normalization, and spatial smoothing (Whitfield-Gabrieli and Nieto-Castanon, 2012). The first five and last four volumes of the rs-fMRI time series were discarded to avoid signal fluctuations. All rs-fMRI images were realigned to the first volume of the time series. The outliers were detected using the artifact detection tool (ART) implemented in CONN. To identify potential outliers with larger motion, a displacement threshold of 0.5 mm was chosen with a threshold of global rs-fMRI signal scaled to standard deviation (z-score) of 3. Data points exceeding either threshold were identified as outliers. All rs-fMRI images were registered to anatomical T1 images, and then normalized onto the standard MNI space. The gray matter, white matter, and cerebrospinal fluid (CSF) tissue regions were then segmented using the default preprocessing step in CONN. The pre-processed rs-fMRI images were then smoothed with a Gaussian kernel of 6 mm full width half maximum (FWHM). After spatial processing, temporal processing steps were employed to remove the unwanted physiological noise. The nuisance signal components derived from white matter and CSF regions, estimated subject-motion parameters, a variable number of noise components found when identifying outliers were used as the temporal confounding factors and were removed separately using ordinary least squares (OLS) regression. After nuisance regression, linear detrending and despiking were also performed, followed by band-pass filtering ranging from 0.01 to 0.1 Hz.

### Identification of Resting State Networks and Regions of Interests

To identify RSNs, we used constrained group independent component analysis (CG-ICA), which is a spatially constrained semi-blind spatial ICA algorithm (Lin et al., 2010). The CG-ICA algorithm uses the prior spatial information of the desired source signals, and enables more robust detection of RSNs whose spatial locations have been previously identified (Goulden et al., 2014). Similar to previous studies (Goulden et al., 2014; Chand et al., 2017), the RSNs defined by Shirer et al. (2012) were used for prior spatial location. Shirer et al. (2012) identified 90 Regions of Interest (ROIs) across 14 intrinsic connectivity networks covering most of the gray matter structures. Within these 90 ROIs, 11 were identified for the dorsal attention network (DAN), 19 for the default mode network (DMN), 12 for the executive control network (ECN), and 19 for the salience network (SAN). These identified ROIs were combined and used as the prior spatial constraints for CG-ICA analysis in this study. The CG-ICA analysis was carried out using Group ICA of fMRI Toolbox (GIFT)^2^ (Calhoun et al., 2001). The four RSNs including DAN, DMN, ECN and SAN were obtained by running the semi-blind infomax algorithm in GIFT toolbox on the preprocessed resting state data. Figure 1B depicts the four representative RSNs from all subjects.

The group level spatial t-maps were computed using GIFT toolbox. To identify the precise center locations of the RSN clusters (e.g., medial prefrontal cortex, posterior cingulate cortex, etc.), the results were loaded into CONN’s result explorer. The T and k threshold was empirically chosen in CONN so that the RSNs are visually the same to the RSN map displayed by GIFT toolbox. A threshold of T (t-maps value) > 14 and k (cluster size) > 100 was applied, identifying 5 clusters for DAN, 5 clusters for DMN, 10 clusters for ECN and 8 clusters for SAN. Among these identified clusters, the regions which have been frequently reported in the four RSNs in the literature were selected for further FC analysis, as listed at the end of this paragraph and shown in Supplementary Figure 1. Subsequently, spherical ROIs with radius of 6 mm centering at the local maxima of the clusters were created, which is similar to previous methods (Li et al., 2013; Kuo et al., 2019; Wang et al., 2020). These ROIs were used to calculate intra- and inter-network FCs.

As shown in Table 1 and Figure 1, a total of four brain networks including 19 ROIs were selected after agreement by a panel discussion (LWK, SA, SMH, and MCT). The network details and nomenclature of the corresponding ROIs are described below:

**TABLE 1:** Anatomical locations and labels for regions involving resting-state networks in the current study.

Resting-state network	*X*	*Y*	*Z*	Label	Anatomical locations
Dorsal attention network (DAN)	−26	−66	50	LIPS	Left inferior parietal sulcus
	28	−62	48	RIPS	Right inferior parietal sulcus
	−48	8	32	LIFG	Left inferior frontal gyrus
	50	8	28	RIFG	Right inferior frontal gyrus
Default mode network (DMN)	6	52	10	MPFC	Medial prefrontal cortex
	0	−56	24	PCC	Posterior cingulate cortex
	−44	−68	28	LIPL	Left inferior parietal lobule
	46	−64	32	RIPL	Right inferior parietal lobule
Executive control network (ECN)	−48	−56	36	LPPC	Left posterior parietal cortex
	52	−50	42	RPPC	Right posterior parietal cortex
	−42	22	44	LDLPFC	Left dorsolateral prefrontal cortex
	42	16	48	RDLPFC	Right dorsolateral prefrontal cortex
Salience network (SAN)	−4	18	38	ACC	Anterior cingulate cortex
	−30	42	26	LMFG	Left middle frontal gyrus
	28	48	22	RMFG	Right middle frontal gyrus
	−60	−34	28	LSMG	Left supramarginal gyrus
	60	−36	30	RSMG	Right supramarginal gyrus
	−46	12	−4	LAI	Left anterior insula
	42	14	−4	RAI	Right anterior insula

(i) Dorsal Attention Network (DAN) (Jimenez et al., 2016; Zhou et al., 2018): Four ROIs, including the bilateral inferior parietal sulcus (LIPS and RIPS) and inferior frontal gyrus (LIFG and RIFG).
(ii) Default Mode Network (DMN) (Taghia et al., 2017; Zhou et al., 2018; Wang et al., 2020): Four ROIs, including the medial prefrontal cortex (MPFC), posterior cingulate cortex (PCC), and bilateral inferior parietal lobule (LIPL and RIPL).
(iii) Executive Control Network (ECN) (Sridharan et al., 2008; Taghia et al., 2017; Chen et al., 2019; Wang et al., 2020): Four ROIs, including the bilateral posterior parietal cortex (LPPC and RPPC) and dorsolateral prefrontal cortex (LDLPFC and RDLPFC).
(iv) Salience Network (SAN) (Taghia et al., 2017; Zhou et al., 2018; Chen et al., 2019; Cai et al., 2020; Wang et al., 2020): Seven ROIs, including the anterior cingulate cortex (ACC) and bilateral middle frontal gyrus (LMFG and RMFG), supramarginal gyrus (LSMG and RSMG), and anterior insula (LAI and RAI).

The rationale for choosing these four RSNs was primarily based on their potential role in apathy formation as reported in previous studies (Levy and Dubois, 2006).

### Analyses of Network Connectivity

The voxel-wise rs-fMRI time series within each ROI (a total of 19 ROIs for 4 RSNs) was averaged and used for the analyses of regional rs-fMRI activity, intra-RSN connectivity and inter-RSN connectivity. To examine the intra- and inter-network connectivity, the ROI-to-ROI connectivity was estimated as the Pearson’s correlation coefficient between the averaged time courses in the ROIs. Therefore, a 19 × 19 connectivity matrix was obtained for each subject, and used to evaluate the intra- and inter-network connectivity (Supplementary Figure 2). The relationships among networks were visualized using Circos software (Krzywinski et al., 2009).^3^

### White Matter Hyperintensities Volume Assessment

We performed automatic WMH segmentation by using the lesion growth algorithm (LGA) (Schmidt et al., 2012) as implemented in the lesion segmentation tool (LST 3.0.0)^4^ for SPM. This algorithm utilizes T1 images and FLAIR images together to estimate the lesion probability of white matter. A pre-chosen initial threshold (κ) of 0.1 was used after visual inspection of the segmentation results. To calculate the total volume of WMH, we used LST’s default settings of probability threshold of 0.5 (probability of a voxel being WMH). Representative result of the WMH segmentation across three groups was presented in Supplementary Figure 3.

### Statistical Analysis

Analysis of variance (ANOVA) and the chi-square test were used to detect the statistical significance of between-group differences in basic information, neuropsychological symptoms, and RSN analysis. Analysis of covariance (ANCOVA) with the Bonferroni method as the *post hoc* test was used to detect statistical significance by controlling for age, education, and MMSE as potential covariates. Factor analysis was used to derive the main factors responsible for AES scores. Pearson’s correlation test was used to examine the relationships between AES_T and its factors with clinical/rs-fMRI parameters. Linear regression analysis with a stepwise regression procedure was used to determine the associations between AES_T and clinical parameters. In the stepwise linear regression analysis, the *p*-values for entry and removal during the stepping method were set as 0.05 and 0.10, respectively, using the probability of F. All statistical tests were performed using SPSS software version 19 (IBM, Armonk, New York). A *p*-value less than 0.05 was considered to be statistically significant.

## Results

### Basic Information and Screening for Neuropsychological Symptoms

Table 2 shows the basic information of all participants. Both the AD and SIVD groups were significantly older than the NC group (*p* < 0.001∼0.002). The education level of the SIVD group was significantly lower than that of the NC group (*p* = 0.010). In systemic cerebral vascular factor assessments, the SIVD group had higher HIS and Fazekas scale scores (all components and total) than the AD and NC groups (*p* < 0.001). The AD group had a higher periventricular component score of the Fazekas scale than the NC group (*p* < 0.020). Consistently, SIVD group had higher WMH volume than the other two groups (*p* = 0.003∼0.014). There were no significant differences in all indices of demographics, global cognition status, and social background between the SIVD and AD groups (*p* = 0.051∼0.872). Most of the participants lived in the community, and only one AD patient resided in a care center.

**TABLE 2:** Basic information among subjects with SIVD, AD, and NC.

	SIVD	AD	NC		
	*N* = 23	*N* = 34	*N* = 23	*F*	*P*
**Demographic**					
Age (year-old)	73.2 (7.55)	77.8 (6.51)	65.7 (7.42)	***19.861***	**<*0.001*^bc^****
Education (years)	7.4 (2.82)	8.2 (3.67)	10.2 (3.32)	***4.643***	***0.018*^b^****
Duration (years)	2.3 (2.00)	3.2 (1.89)	0.0 (0.00)	***25.514***	** < *0.001*^bc^****
Gender (Male/Female)	13/10	17/17	12/11	-	0.888
Handedness (Right/Left)	22/1	33/1	23/0	-	0.777
**Systemic and cerebral vascular factors**					
Hachinski Ischemic Scale	9.4 (2.48)	1.7 (1.32)	1.0 (0.90)	***186.955***	**<*0.001*^ab^****
Fazekas scale – Periventricular WMH	1.9 (0.36)	1.0 (0.60)	0.5 (0.59)	***38.531***	**<*0.001*^abc^****
– Deep WMH	1.9 (0.51)	0.6 (0.58)	0.5 (0.51)	***45.620***	**<*0.001*^ab^****
– Total	3.8 (0.62)	1.6 (0.94)	1.0 (0.90)	***68.307***	**<*0.001*^ab^****
WMH volume (mL)	8.9 (6.67)	4.6 (4.07)	3.3 (6.45)	***6.455***	***0.003*^ab^****
**Global cognition**					
Clinical dementia rating_ sum of box	5.5 (4.13)	4.4 (2.82)	0.0 (0.00)	***18.973***	**<*0.001*^bc^****
Cognitive abilities screening instrument	61.9 (18.19)	65.5 (16.91)	87.8 (4.79)	***21.025***	**<*0.001*^bc^****
Mini-mental state examination	19.0 (6.22)	21.7 (4.29)	27.8 (1.60)	***24.309***	**<*0.001*^bc^****
**Social background**					
Marital status (1 = married; 2 = unmarried or divorced; 3 = widowed)	17/1/5	24/0/10	17/2/4	-	-
Supportive System (1 = living alone; 2 = with family; 3 = care center)	0/23/0	6/27/1	2/21/0	-	-
Religious status (1 = Dao/Buddha; 2 = Christian/Catholic; 3 = none)	21/1/0	31/0/2	19/3/1	-	-

*SIVD, Subcortical ischemic vascular disease; AD, Alzheimer’s disease; NC, Normal Cognition; WMH, White matter hyperintensities. Data presented as Mean (Standard deviation) unless stated elsewhere. Comparisons were made by using the Analysis of variance (ANOVA) and Chi-square test where appropriate. P-values < 0.05 are boldly italicized. F-values of ANOVA were reported. P-values < 0.05 resulting from post hoc Tukey analyses were as follows:*

*^a^between SIVD and AD,*

*^b^between SIVD and NC,*

*^c^between AD and NC.*

**FIGURE 2:**
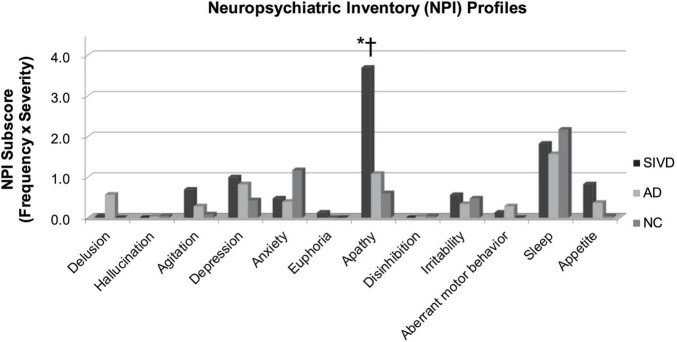
Comparisons of neuropsychiatric symptoms. SIVD, Subcortical ischemic vascular disease; AD, Alzheimer’s disease; NC, Normal Cognition. Comparisons were made by using the Analysis of variance (ANOVA). *P*-values < 0.05 resulting from *post hoc* Tukey analyses were as follows: *: between SIVD and AD, †: between SIVD and NC.

Figure 2 shows the comparisons of each individual symptom of the NPI by group. The apathy score in the SIVD group was significantly higher than that in either the AD or NC group (*p* < 0.001). The SIVD group still had a higher apathy score than the other two groups after controlling for age, education, and MMSE (*p* = 0.001∼0.020). The between-group differences were not significant regarding the other NPI symptoms (*p* = 0.122∼0.995).

### Factor Analysis of Apathy Evaluation Scale

Table 3 shows the results of exploratory factor analysis aimed at determining the main factors for the construct of the AES. Principal axis factor analysis revealed three factors with an eigenvalue > 1, and therefore a three-factor model was selected after examining the scree plot. Communalities among all 18 items of the AES ranged from 0.50 to 0.88, suggesting that all items contributed a considerable effect to the determined factors. The rotated component matrix based on varimax rotation showed no correlations above 0.7, hence primarily excluding the issue of multicollinearity. The Kaiser-Meyer-Olkin measure of sampling adequacy (0.926) and Bartlett’s test of sphericity (*p* < 0.001) suggested that the factor analysis was appropriate for the current data. These factors were defined as Initiation (AES_I), Motivation (AES_M), and Sociality (AES_S) based on the main property of their corresponding items from the AES questionnaire. The eigenvalues of these three factors were 10.98 (AES_I), 1.35 (AES_M), and 1.16 (AES_S), and the variances were 61.0 (AES_I), 7.5 (AES_M), and 6.5 (AES_S), therefore accounting for 75% of the variance in the current cohort.

**TABLE 3:** Factor loadings of the Apathy Evaluation Scale.

Items	Factor loading		
	Initiation	Motivation	Sociality
Effort (6)	0.85		
Insight (15)	0.82		
Less concerned (11)	0.79		
Someone has to tell her/him to do (10)	0.77		
Getting things done during the day is important (16)	0.67		
Getting excited on good thing (14)	0.63		
Get things done (2)	0.62		
Initiative (17)	0.60		
Learning new things (5)		0.85	
New experience (4)		0.80	
Spend time doing things (9)		0.73	
Motivation (18)		0.69	
Interest (1)		0.67	
Approach life with intensity (7)		0.66	
Get things started (3)		0.66	
Seeing a job through to the end (8)		0.60	
Getting together with friends (13)			0.89
Friend (12)			0.86

*Each factor from the AES was reported as question (question number) in this table. The factor loadings were sorted by size, and those with value less than 0.6 were not displayed.*

Table 4 shows comparisons of behavioral and psychological symptoms of dementia in all three groups. After controlling for age and education, the total NPI, BAI and BDI scores were insignificant across groups. Of note, AES_T in the SIVD group was significantly higher than in the AD (*p* = 0.007) and NC (*p* < 0.001) groups. Further analysis of the three main AES factors revealed that the SIVD group had a significantly higher AES_I value than the AD (*p* = 0.001) and NC (*p* < 0.001) groups, while there were no significant between-group differences in AES_M and AES_S (*p* = 0.159∼1). After additionally controlling for MMSE, marginal differences of SIVD > AD were only found for AES_T (*p* = 0.072) and AES_I (*p* = 0.061).

**TABLE 4:** Comparisons of targeted neuropsychiatric symptoms and apathy main factors among patients with SIVD, AD, and NC.

	SIVD	AD	NC		
	*N* = 23	*N* = 34	*N* = 23	*P*	Controlled by age and education
Neuropsychiatric Inventory—total	9.3 (8.65)	5.9 (8.23)	4.3 (5.31)	0.089	n.s.
Beck Anxiety Inventory	4.8 (7.38)	4.0 (7.03)	7.4 (9.10)	0.293	n.s.
Beck Depression Inventory	8.4 (11.53)	5.9 (7.93)	7.9 (6.74)	0.552	n.s.
Apathy Evaluation Scale—total	45.2 (14.69)	34.4 (13.40)	29.2 (9.34)	**<*0.001*^ab^****	***SIVD* > *AD; SIVD* > *NC***
—Initiation	0.63 (1.29)	−0.08 (0.76)	−0.51 (0.57)	**<*0.001*^ab^****	***SIVD* > *AD; SIVD* > *NC***
—Motivation	0.32 (0.97)	−0.16 (0.93)	−0.07 (1.07)	0.168	n.s.
—Sociality	0.09 (1.05)	0.09 (1.01)	−0.22 (0.92)	0.437	n.s.

*SIVD, Subcortical ischemic vascular disease; AD, Alzheimer’s disease; NC, Normal Cognition; ns, non-significant. Data presented as Mean (Standard deviation) unless stated elsewhere. Comparisons were made by using the Analysis of variance (ANOVA) and Analysis of covariance (ANCOVA). P-values < 0.05 are boldly italicized. In ANOVA, P-values < 0.05 resulting from post hoc Tukey analyses were as follows:*

*^a^between SIVD and AD,*

*^b^between SIVD and NC,*

*^c^between AD and NC. In ANCOVA, significant results from the Bonferroni method as the post hoc test were reported. No between-group difference was noted on controlling age, education, and the Mini-Mental State Examination.*

### Association Between Clinical Factors and Apathy Formation

Given a significant correlation between WMH volume and the Fazekas scale (*r* = 0.589, *p* < 0.001), WMH volume in addition to clinical parameters including age, education, gender, BAI, BDI, and CDR_SOB were selected to enter into a stepwise linear regression analysis. Table 5 shows the significant predictors among these variables for AES, suggesting that CDR_SOB (*p* < 0.001), WMH volume (*p* = 0.006), and BDI (*p* = 0.015) constituted the main factor in predicting apathy severity, accounting for 50% of variance.

**TABLE 5:** Summary of the linear regression analysis in exploring clinical predictors for apathy severity.

Model		Unstandardized coefficients		Standardized coefficients			Summary of model				
		*B*	Standard Error	Beta (b*)	*t*	*p*	*R* ^2^	Adjusted *R*^2^	△*R*^2^	△*F*	*P*-value of △*F*
1	Constant	26.358	1.780		14.811	< 0.001	0.425	0.417	0.425	56.867	< 0.001
	CDR_SOB	2.641	0.350	0.652	7.541	< 0.001					
2	Constant	24.469	1.851		13.217	< 0.001	0.475	0.461	0.050	7.192	0.009
	CDR_SOB	2.322	0.357	0.573	6.499	< 0.001					
	WMH volume	0.556	0.207	0.236	2.682	0.009					
3	Constant	22.098	2.026		10.906	< 0.001	0.515	0.496	0.040	6.244	0.015
	CDR_SOB	2.333	0.346	0.576	6.752	< 0.001					
	WMH volume	0.572	0.201	0.243	2.851	0.006					
	BDI	0.325	0.130	0.201	2.499	0.015					

*The independent variables entered into the stepwise linear regression model included age, education, gender, the Beck Anxiety Inventory, the Beck Depression Inventory (BDI), Clinical Dementia Rating sum of box (CDR_SOB), and white matter hyperintensities (WMH) volume. None of the first-order correlations were above 0.70 (or below -0.70), therefore generally excluding the issue of multicollinearity. Variables including age, education, gender, and the Beck Anxiety Inventory were finally removed.*

### Between-Group Comparisons of Functional Connectivity

**FIGURE 3:**
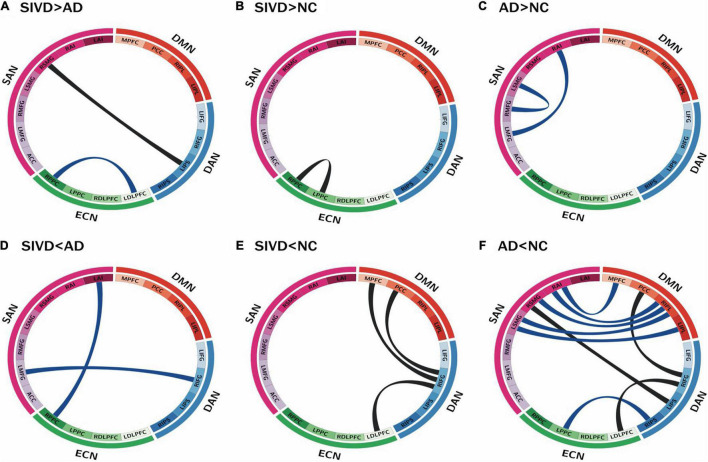
Connectogram of functional connectivity by groups. Four networks are remarked by their corresponding sub-regions and distinct colors: blue for DAN, orange for DMN, green for ECN, and purple for SAN. Each ribbon shows the functional connectivity between the sub-regions where the ends of the ribbon touches. Between-group comparisons after controlling for age, education, and global cognition were as labeled: **(A)** SIVD > AD **(B)** SIVD > NC **(C)** AD > NC **(D)** SIVD < AD **(E)** SIVD < NC **(F)** AD < NC. Black and blue band represent connectivity differences with *p*-values < 0.01 and < 0.05, respectively. SIVD, Subcortical ischemic vascular disease; AD, Alzheimer’s disease; NC, normal cognition. DAN, dorsal attention network; DMN, default mode network; ECN, executive control network; SAN, salience network; L/RIPS, left/right inferior parietal Sulcus; L/RIFG, left/right inferior frontal gyrus; MPFC, medial prefrontal cortex; PCC, posterior cingulate cortex; L/RIPL, left/right inferior parietal lobule; L/RPPC, left/right posterior parietal cortex; L/RDLPFC, left/right dorsolateral prefrontal cortex; ACC, anterior cingulate cortex; L/RMFG, left/right middle frontal gyrus; L/RSMG, left/right supramarginal gyrus; L/RAI, left/right anterior insula. “>” and “<” represent “significantly higher” and “significantly lower,” respectively.

Figure 3 illustrates significant between-group differences in FC after controlling for age, education, and MMSE. In SIVD-AD comparisons (Figures 3A,D), the SIVD group had a greater FC than the AD group in two connections, i.e., RSMG-LIPS and RPPC-LDLPFC (*p* = 0.009∼0.039), but lower FC in the other two connections, i.e., RIFG-LMFG and RPPC-LAI (*p* = 0.027∼0.039). These four connections participated in the DAN, SAN, and ECN, and three of them were related to inter-RSN connections. In SIVD-NC comparisons (Figures 3B,E), the SIVD group exhibited greater FC in the RPPC-LPPC connection (*p* = 0.008), and lower FC in three connections which were all related to the DAN (i.e., RIFG-MPFC, RIFG-PCC, and RIFG-LDLPFC (*p* = 0.006∼0.009).

In comparisons of FC with the NC group, both the AD and SIVD groups appeared to have several higher FC connections but more lower FC connections (Figures 3B,C,E,F), and the AD group had more connections with lower FC than the SIVD group (Figures 3E,F). The connections with higher FC were all categorized as intra-RSNs (i.e., ECN in the SIVD group in Figure 3B; SAN in the AD group in Figure 3C), whilst the connections with lower FC were all categorized as inter-RSNs (Figures 3E,F). In the SIVD group, connections with lower FC were identified in the DAN-DMN and DAN-ECN. In the AD group, connections with lower FC were identified between the DAN and the other three RSNs, and DMN-SAN. Notably, significant FC changes with *p*-values < 0.01 (depicted as black bands in Figures 3E,F) were all related to the DAN, including (i) RIFG-MPFC, RIFG-PCC, and RIFG-LDLPFC connections (*p* = 0.006∼0.009; SIVD < NC) and (ii) RIFG-LDLPFC, RIFG-PCC, and LIPS-RSMG connections (*p* = 0.002∼0.007; AD < NC). Both the SIVD and AD groups exhibited significantly lower FCs in RIFG-PCC and RIFG-LDLPFC connections compared to the NC group.

### Correlations Between Apathy Evaluation Scale-Clinician Version Factors and Shared Functional Connectivity in the Subcortical Ischemic Vascular Disease and Alzheimer’s Disease Groups

**FIGURE 4:**
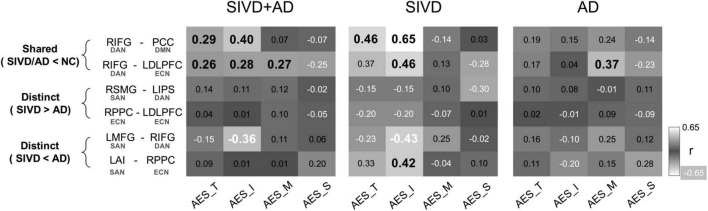
Correlations between brain connectivity with apathy factors. Correlations between AES factors and connectivity between-group differences were displayed. The results were reported by the Pearson correlation coefficient, and values with significance were labeled by bold italics. SIVD, Subcortical ischemic vascular disease; AD, Alzheimer’s disease. AES_T/I/M/S, Apathy Evaluation Scale total scores/Initiation/Motivation/Sociality. RIFG, right inferior frontal gyrus; PCC, posterior cingulate cortex; LDLPFC, left dorsolateral prefrontal cortex; RSMG, right supramarginal gyrus; LIPS, left inferior parietal sulcus; RPPC, right posterior parietal cortex; LMFG, left middle frontal gyrus; LAI, left anterior insula. DAN, dorsal attention network; DMN, default mode network; ECN, executive control network; SAN, salience network. “>” and “<” represent “significantly higher” and “significantly lower,” respectively.

In order to explore the clinical significance in terms of FC differences in the SIVD, AD, and NC groups, the correlations between the identified FC differences and AES factors (i.e., AES_I, AES_M, and AES_S) were analyzed (Figure 4). For the shared FC differences in the SIVD and AD groups compared to the NC group, correlation analysis of the SIVD and AD groups combined showed a significant correlation between the FC in RIFG-PCC and AES-I (*r* = 0.40, *p* = 0.002) but not AES_M or AES_S (*p* = 0.59∼0.62). The FC in RIFG-LDLPFC was significantly correlated with AES_I (*r* = 0.28, *p* = 0.038) and AES_M (*r* = 0.27, *p* = 0.046), but only marginally with AES_S (*r* = -0.25, *p* = 0.058). These two FC connections, RIFG-PCC and RIFG-LDLPFC, were significantly correlated with AES_T (*r* = 0.26∼0.29, *p* = 0.027∼0.046).

### Correlations Between Apathy Evaluation Scale-Clinician Version Factors and Distinct Functional Connectivity Between the Subcortical Ischemic Vascular Disease and Alzheimer’s Disease Groups

With regards to the identified FC differences between the SIVD and AD groups, three significant correlations between AES factors were identified, and all of which were related to the SAN. Furthermore, all of the FC differences were SIVD < AD, and their significant correlations were confined to AES_I. In analysis of the SIVD and AD groups combined, the FC in the RIFG-LMFG connection was significantly inversely correlated with AES_I (*r* = -0.36, *p* = 0.006). On analyzing the SIVD and AD groups separately, the FC in the LMFG-RIFG (*r* = -0.43, *p* = 0.041) and LAI -RPPC (*r* = 0.42, *p* = 0.048) connections showed significant correlations with AES_I in the SIVD group, but no significant correlations were identified in the AD group. None of the connections with FC differences between the SIVD and AD groups showed significant correlations with AES_T (*p* = 0.129∼0.925).

## Discussion

In this study, we detailed the clinical and psychological structures of apathy in line with FC changes in patients with SIVD and AD. In comprehensive analysis of the four related RSNs, both intra- and inter-RSN FCs showed dissociable FC properties and FC-apathy correlations. Comparisons between SIVD/AD and NC groups indicated that intra-RSN differences involved higher FC, whereas inter-RSN differences were of lower FC. Among the inter-RSN connections with lower FC, the SIVD and AD groups had two shared connections compared to the NC group, RIFG (in the DAN)—PCC (in the DMN) and RIFG (in the DAN)—LDLPFC (in the ECN). Contrary to the insignificant correlations between AES and connections with FC showing SIVD > AD, two distinct connections with FC showing SIVD < AD were found in LMFG-RIFG and LAI-RPPC, and both were related to the SAN and correlated with AES structure to a certain degree. Further in-depth analysis revealed that these SAN-related connections had a convergent correlation with AES-I in the SIVD group, in contrast to the divergent correlation between the shared connection with AES and its factors. Taken together, our results highlight the key role of the DAN, since RIFG was associated with all of the significantly divergent connections identified in both the SIVD and AD groups, and support the role of the SAN in initiation of voluntary goal-directed action.

Both patients with SIVD and AD have been reported to exhibit cerebral macro/microstructural changes within the gray (Yi et al., 2012; Gong et al., 2017) and white matter (Tu et al., 2017b). While damage within the white matter has been inherently regarded to be responsible for the disconnection syndrome, gray matter insults can also contribute or interact with the process of cerebral fiber disconnection. At the RSN level, the SIVD and AD groups in this study shared two connections with lower FC in RIFG—PCC and RIFG—LDLPFC, which showed divergent correlations with the total scores and factors of AES. Previous rs-fMRI and electrophysiological studies have highlighted the role of IFG in integrating the DAN and ventral attention network (Asplund et al., 2010), or in serving as a general control across cognition, behavior, and emotion (Tops and Boksem, 2011). Our rs-fMRI results further elaborate on the concept of the disconnection syndrome in view of functional interactions amongst multiple RSNs, and suggest that inter-RSN connections should be considered as a task-related functional ensemble despite the physical distance among these cortical regions. For instance, the DAN could serve as a critical ensemble, as both the SIVD and AD groups shared connections with FC lower than that in the NC group within the DAN, particularly those related to RIFG. The DAN is regarded to be a unique vector for attention-demanding tasks owing to its role in cognitive control through widespread outbound connections. Consistently, in the present study, the DAN also exhibited divergent connections toward the DMN and ECN across patients, and also to the SAN in the AD patients. These findings suggest that action switch in response to external stimuli could substantially rely on coordinated intrinsic activity among RSNs, such as the DMN (Joo et al., 2017) and ECN (Munro et al., 2015; Joo et al., 2017), which are also related with apathy formation. However, caution should be taken when interpreting the biological implications regarding a greater number of significant inter-RSN connections in patients with AD than SIVD as compared to subjects with NC, as the findings could be an epiphenomenon reflecting *a priori* knowledge that AD-related pathology is primarily located within the gray matter (Montine et al., 2012). A greater number of irrelevant FCs may also attenuate the composite output in effect as the signal-to-noise ratio decreases (Levy and Dubois, 2006). Therefore, the number *per se* may not be equivalent to the impact of FCs.

It is also worth pointing out that the significant correlations between the SAN and AES-I bridged RSN-specific alterations to apathy formation. This suggests that SAN components including LMFG and LAI are functional hubs for apathy, which is generally consistent with published articles that have reported associations between structural damage within the left hemisphere and apathy formation (Kim et al., 2011; Tunnard et al., 2011; Tu et al., 2017a). Contrary to the FCs of SIVD > AD, the FCs of SIVD < AD were all related to the SAN with significant correlations confined to AES_I, indicating that a disturbed SAN could be associated with impaired auto-activation processes among patients with dementia, particularly those with SIVD. Several studies using other rs-fMRI methods have reported interesting parallels that together mirror SAN alterations in apathy formation. Using seed-based analysis in which both inter- and intra-RSN FCs were summed together (Joel et al., 2011), significantly increased FCs in RAI-RDLPFC and RAI-RPCC were found in patients with geriatric depression and high apathy compared to those with geriatric depression but low apathy (Yuen et al., 2014). Two other studies using topology both reported lower local efficiency within the ACC as a surrogate for an altered SAN in response to apathy formation (Onoda and Yamaguchi, 2015; Tumati et al., 2020). Saliency detection represents a critical process in which the encoded value of a stimulus facilitates an action toward or away from the stimulus (Berridge et al., 2009). Therefore, we hypothesize that the SAN serves as an important bottom-up processor which could be responsible for apathy formation, as it normally governs dedicated switch-on and off of other RSNs according to the saliency of upcoming stimuli. This putative role of the SAN may assist in tailoring intervention programs for patients with dementia and apathy. Further studies are needed to investigate whether the laterality effect can be generalized across all dementia subtypes.

We also determined the significant predictors for apathy severity among clinical parameters that are commonly inter-related. Consistent with previous studies (Jonsson et al., 2010; Tu et al., 2017a), dementia staging and WMH contributed considerable variance to apathy. While the overall 50% explanatory variance in our model was statistically significant, it also highlights the clinical need for additional biomarkers to improve the precision in capturing apathy. Apathy has been conceptually categorized as “auto-activation,” “cognitive,” and “emotional-affective” subtypes (Levy and Dubois, 2006), where the “auto-activation” deficit has been proposed to be a failure of internalized guidance that facilitates activities within the prefrontal cortex and basal ganglia. In the factor analysis of our study, questions related to AES-I such as *“S/he puts little effort into anything”* also attempt to measure subject’s willingness to work, ability on valuing the action (Le Heron et al., 2018), or contrast goal-directed behavior between internally and externally driven dynamics (Levy and Dubois, 2006). The LMFG that was found to be a significant neuronal substrate for apathy in our results is part of the prefrontal cortex, and the prefrontal cortex has been postulated to be a center in which initiation, maintenance and shift of action are highly reliant (Levy and Dubois, 2006). The LAI has also emerged as a significant hub, probably based on its structural connections with the amygdala (Ghaziri et al., 2018) as well as its role in the SAN (Taghia et al., 2017; Zhou et al., 2018; Chen et al., 2019; Cai et al., 2020; Wang et al., 2020).

The strengths of the current study include the interdisciplinary study design comparing apathy severity, FCs, and their associations with two dementia subtypes. The factorized components of apathy were mapped onto several dissociable yet inter-related RSNs. Our current work could have clinical implications. First, the structure of apathy, which can be factorized in psychometrics, can also be described and analyzed on a multi-RSN level. This indicates the potential of fMRI to detect and/or follow the presentations of apathy by deploying a proper multi-RSN paradigm. Second, as existence of apathy has negative impact to clinical outcomes from both cross-sectional (Parrotta et al., 2020) and longitudinal (van der Linde et al., 2017) studies, a personalized intervention therapeutics could be tailored by specifying psychometric factors and altered RSNs, as it might bring immediate and long-term benefit for dementia patients with apathy. However, several limitations should also be addressed. First, the sample size was limited and included patients with only two dementia subtypes. Although we performed the analysis after considering the possibility of mixed dementia in addition to potential effects from demographic and cognitive confounders, future studies with a larger sample size and other dementia subtypes are needed to generalize the disconnection mechanism. Second, although we discussed the effects of FC by parceling intra- and inter-RSN interactions, FC revealed by inter-regional temporal correlations under a spatial ICA paradigm may still contain signals merged with some other irrelevant RSNs (Smith et al., 2012), thereby confounding the interpretation about whether the targeted FC was operationally effective. It may be a challenge to reconcile opposite intra- and inter-RSN FC changes across dementia groups compared to subjects with NC due to the poly-synaptic properties of these FCs. Other state-of-the-art fMRI methods could provide complementary information, such as temporal ICA (Smith et al., 2012), dynamic FC network analysis (Fu et al., 2019), and variable algorithms aimed at evaluating effective connectivity (Horwitz et al., 2005). Third, the current study evaluated the FCs across four principal RSNs, in which the connectograms were converged to provide a scale that could be used for both clinical and research needs. Other FC differences from regions not discussed in this study are possible, which could have shown apparent correlations with apathy structure. Inclusive analysis of whole brain connectivity (Veer et al., 2010) could provide more information in future studies. Lastly, as it remains a great challenge for the current study to further specify process related to initiating a behavior, such as auto-activation and effort/reward-based decision-making, deploying task-based fMRI along with a dedicate psychological experiment may shed light to neurocognitive framework of apathy by detailing its task-specific FCs (Bonnelle et al., 2016).

In summary, the current interdisciplinary research showed that dementia staging, WMH volume, and BDI were the best predictors for apathy severity. Factor analysis operatively derived from AES showed a greater degree of apathy, especially the “Initiation” factor, in patients with SIVD compared to those with AD. This “Initiation” deficit was primarily related to the DAN and SAN, and the shared and distinct FC between SIVD and AD, respectively. Our findings broaden the concept of the disconnection theory through FC interactions and their effects on apathy across multiple RSNs.

## Data Availability Statement

The derived data that support the findings of this study are available on reasonable request from the corresponding author.

## Ethics Statement

The studies involving human participants were reviewed and approved by the Research Ethics Committee of the Taichung Tzu Chi Hospital, Buddhist Tzu Chi Medical Foundation. The patients/participants provided their written informed consent to participate in this study.

## Author Contributions

SA, S-MH, L-WK, and M-CT: study concept and design, analysis, interpretation, and drafting the manuscript. M-CT: patient recruitment and clinical examination. Y-HH: neuropsychological test assessment and interpretation. SA and S-MH: graph design. J-JY and C-YL: neuroimaging data collection. All authors contributed in writing the manuscript.

## Conflict of Interest

C-YL was employed by company GE Medical Systems Taiwan, Ltd. The remaining authors declare that the research was conducted in the absence of any commercial or financial relationships that could be construed as a potential conflict of interest.

## Publisher’s Note

All claims expressed in this article are solely those of the authors and do not necessarily represent those of their affiliated organizations, or those of the publisher, the editors and the reviewers. Any product that may be evaluated in this article, or claim that may be made by its manufacturer, is not guaranteed or endorsed by the publisher.

## Footnotes

1
www.nitrc.org/projects/conn

2
http://mialab.mrn.org/software/gift/

3
http://circos.ca/software/

4
https://www.applied-statistics.de/lst.html
