# Supplementary material for: Dissociable Functional Brain Networks Associated With Apathy in Subcortical Ischemic Vascular Disease and Alzheimer’s Disease
Source: Front Aging Neurosci. 2022 Feb 3;13:717037. doi: 10.3389/fnagi.2021.717037 (PMC8851472; doi:10.3389/fnagi.2021.717037)
Supplement: Supplementary file 1 [file Data_Sheet_1.pdf]

Supplemental Material

Dissociable Functional Brain Networks Associated with Apathy in Subcortical Ischemic Vascular Disease and Alzheimer's Disease

Table S1. The inclusion and exclusion criteria for SIVD and AD

**The Inclusion Criteria for SIVD**
(1) cognitive complaints with interference in complex occupational and social activities (2) evidence of subcortical ischemic changes in brain MRI<br>(3) Clinical Dementia Rating (CDR) = 0.5 ~ 1<br>(4) Mini-Mental State Examination (MMSE) score ≤ 26<br>(5) Hachinski Ischemic Scale score (HIS) ≥ 7
**The Inclusion Criteria for AD**
(1) changes in cognition reported by the patient, informant or clinician<br>(2) absence of profound subcortical ischemic change in brain MRI<br>(3) CDR = 0.5 ~ 1<br>(4) MMSE score ≤ 26<br>(5) HIS ≤ 4
**The Exclusion Criteria for SIVD and AD**
(1) state of delirium<br>(2) stroke event within 2 weeks<br>(3) appearance of cortical and/or cortico-subcortical non-lacunar territorial infarcts and watershed infarcts, hemorrhages, signs of normal pressure hydrocephalus, and specific causes of white matter lesions (e.g. multiple sclerosis, sarcoidosis, brain irradiation)<br>(4) derangements in serology tests contributing to cognitive impairment (e.g. abnormal levels of free T4, cortisol, folic acid, vitamin B12, or rapid plasma reagin)<br>(5) severe hearing or visual impairment

Table S2. The Fazekas scales scoring standards

**Periventricular White Matter Hyperintensities (PWMH)**
0 = absent
1 = “caps” or pencil-thin lining
2 = smooth “halo”
3 = irregular periventricular signal extending into the deep white matter
**Deep White Matter Hyperintensities (DWMH)**
0 = absent
1 = punctate foci
2 = beginning confluence
3 = large confluent areas
**Total White Matter Hyperintensities (TWMH)= PWMH+ DWMH**

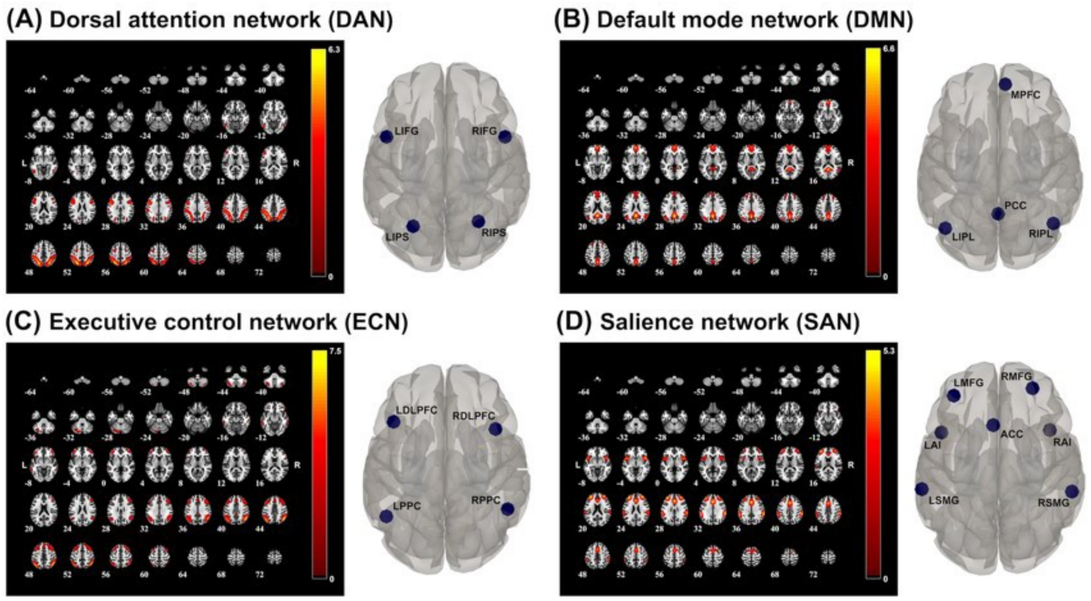

Figure S1. The four main resting state networks in all subjects identified by CG-ICA analysis, including dorsal attention network (DAN), default mode network (DMN), executive control network (ECN), and salience network (SAN). The regions of interest (ROI) selected for functional connectivity analysis are shown. ***Region abbreviations:*** *LIFG/RIFG: left/right inferior frontal gyrus. LIPS/RIPS: left/right inferior parietal sulcus. LIPL/RIPL: left/right inferior parietal lobule. LDLPFC/RDLPFC: left/right dorsolateral prefrontal cortex. LPPC/RPPC: left/right posterior parietal cortex. LMFG/RMFG: left/right middle frontal gyrus. LAI/RAI: left/right anterior insula. LSMG/RSMG: left/right supramarginal gyrus. MPFC: medial prefrontal cortex. ACC: anterior cingulate cortex. PCC: posterior cingulate cortex.*

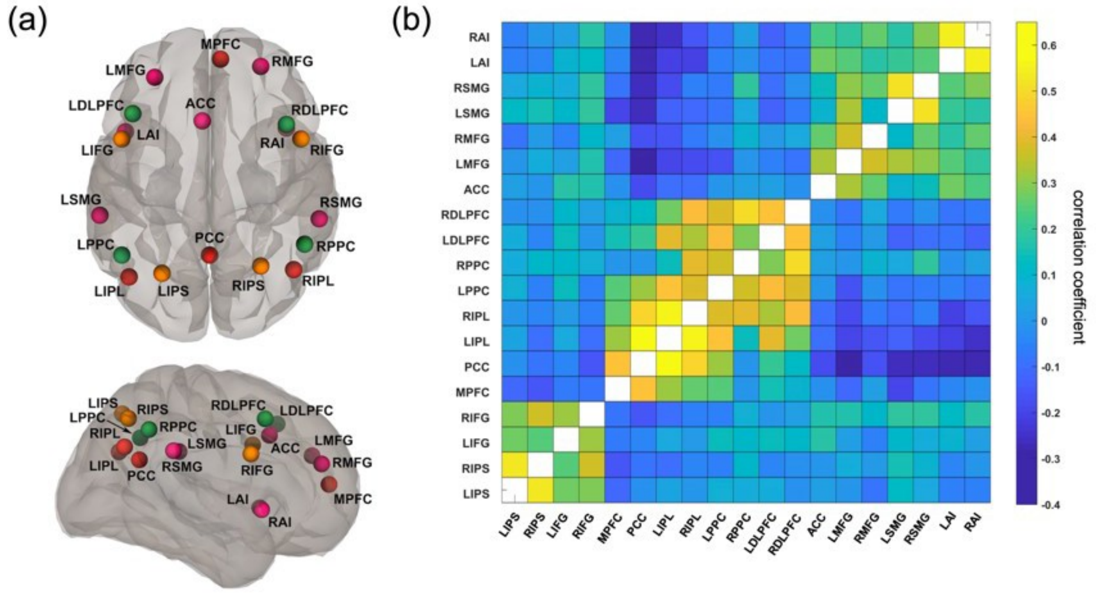

Figure S2. (a) A total of 19 regions of interest (ROI) were selected for functional connectivity analysis. The signal time-series in each ROI was extracted by averaging all of the voxels inside the 6-mm-radius spherical ROI. By calculating the correlation coefficient between the time-series of each two ROIs, a 19x19 connectivity matrix can be obtained for each subject. The averaged connectivity matrix of the normal control group is shown in **(b)**. *Region abbreviations: As listed in Figure S1.*

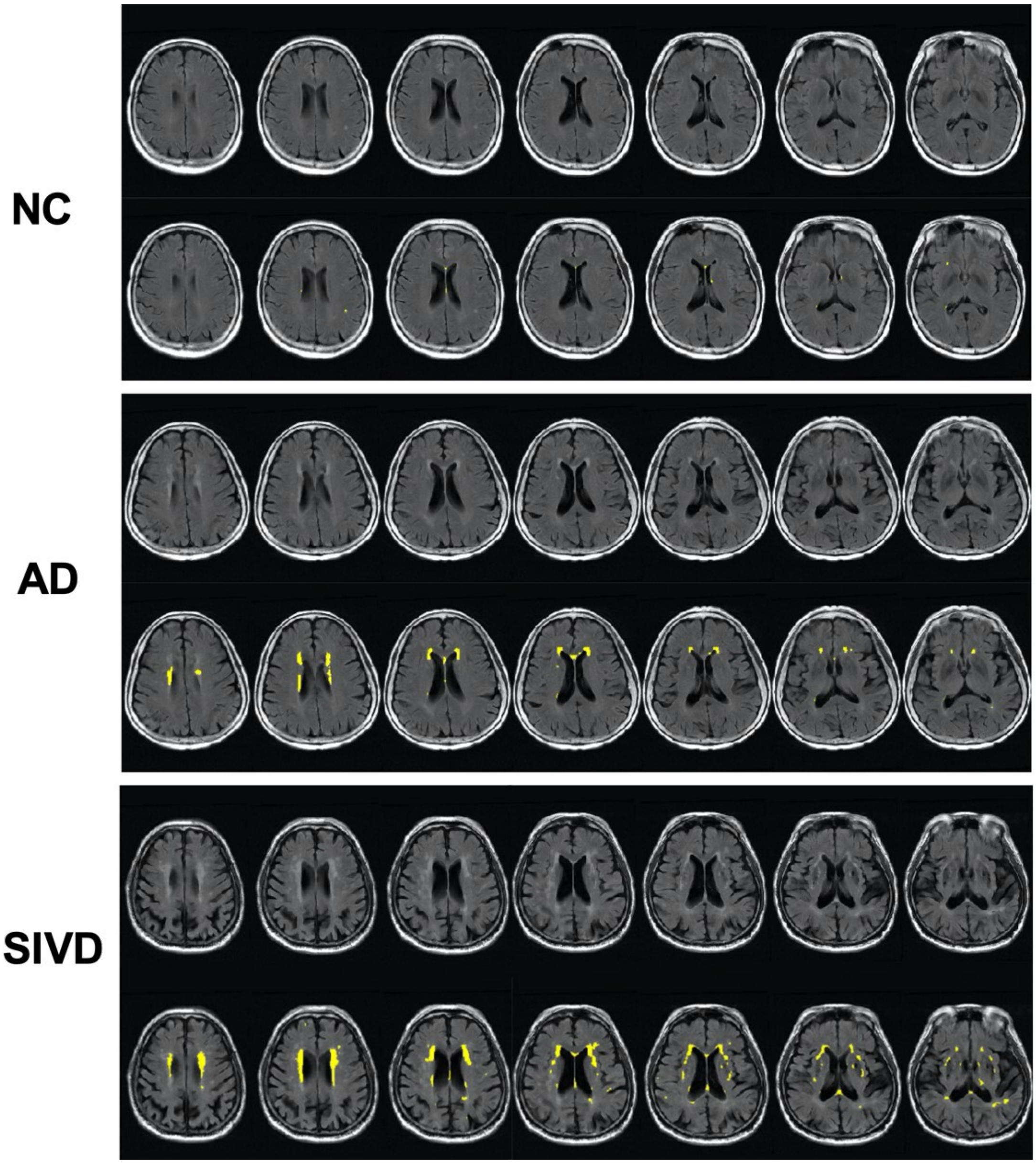

Figure S3. Illustration of volume assessment of white matter hyperintensities by using lesion growing algorithm (LGA) in lesion segmentation toolbox (LST). SIVD: Subcortical ischemic vascular disease; AD: Alzheimer's disease; NC: Normal Cognition.
